# Patient Acceptance and Intention to Use e-Consultations During the COVID-19 Pandemic in the Eastern Province of Saudi Arabia

**DOI:** 10.3389/fpubh.2022.896546

**Published:** 2022-06-30

**Authors:** Arwa Althumairi, Beyan Hariri, Duaa Aljabri, Afnan Aljaffary

**Affiliations:** Health Information Management and Technology Department, College of Public Health, Imam Abdulrahman Bin Faisal University, Dammam, Saudi Arabia

**Keywords:** e-consultations, TAM, COVID-19, trust, usefulness

## Abstract

**Background:**

Over the last decade, the use of digital technology has increased immeasurably and transformed both our personal and professional lives. The medical profession quickly embraced this development, especially after the spread of the COVID-19 pandemic. Medical consultations were transitioned to online settings as a substitute for face-to-face consultations. This exponential acceleration of the use of remote online consultations (e-consultations) was deemed necessary to respond to the impact of the global pandemic. This study identifies the factors that influence actual patient use and the intention to use e-consultations in Saudi Arabia.

**Methods:**

A cross-sectional survey was distributed online via social media platforms targeting the population living in Saudi Arabia from August to December 2020. The questionnaire measured patient perceptions of and attitudes toward utilizing e-consultations using a validated questionnaire informed by the technology acceptance model (TAM). Analyses were performed in SPSS to identify the external factors that influence patients' actual use of e-consultations and to assess the TAM factors (usefulness, social influence, and ease of use) that influence the intention to use e-consultations across both actual users and never-users.

**Results:**

A total of 150 participants completed the questionnaire; the average age was 38 years old, 85% of the participants were females, and 67% reported never using e-consultations. Additionally, motivation, trust, attitude, and social influence were significantly related to participants' intention to use e-consultations.

**Conclusion:**

Participants' trust in and perception of the usefulness of e-consultations were significant factors in their intention to use e-consultation services. Policymakers' attention to those factors could play a role in increasing public acceptance and the use of e-consultations to improve distance medical care.

## Introduction

For several decades, telemedicine has been adopted to provide healthcare services to those in need by ensuring that these services are reached virtually with high quality and efficiency (1–4). Telemedicine is usually used for specific purposes, such as treating and providing e-consultations for patients in rural areas, those with chronic illness, and elderly patients (2, 5). Moreover, in recent years, the use of e-consultations has shown similar outcomes to face-to-face consultations (5, 6). Healthcare management and delivery of care have undergone a transformational change to reduce crowding and distance treatable patients using e-consultations (7–10). Despite advances and uptake in telemedicine, geographical disparities exist, which have become more pronounced during COVID-19 (11). More importantly, user satisfaction and willingness to use e-services are among the essential elements for the success of healthcare policy aims and goals to incorporate technology into healthcare services (12, 13). This is also in line with the Ministry of Health 2030 vision and a new model of care in Saudi Arabia, where adapting technology in the delivery of care is one of the central goals to improve the quality and accessibility of care to the whole population (14). Several studies have been conducted to assess digital health in Saudi Arabia; however, these studies have focused on one e-service (phone calls) or certain health services (dermatology) or were conducted before the COVID-19 pandemic (15–17). This study adds to the literature by identifying the factors influencing actual use and intention to use e-consultations during and after the COVID-19 pandemic. Thus, it contributes important knowledge for policymakers aiming to improve digital health.

In the context of a global pandemic that affected all aspects of life, including hospital visits, and to control the spread of COVID-19, hospitals have restricted visits to only those with urgent conditions. Therefore, the exponential acceleration of the use of virtual services has been deemed necessary to respond to the impact of the pandemic. In particular, e-consultations are an excellent substitute for face-to-face consultations in many settings (16, 18). However, new methods of delivering health using technology could face some resistance due to factors related to trust in the system, lack of understanding its benefits, or digital illiteracy and the difficulty of use (2, 19–21). However, these studies have only assessed certain conditions (19, 20) or specific programs that are tailored to specific hospitals (18).

This study aims to understand the factors influencing the actual use of e-consultations during the COVID-19 pandemic in Saudi Arabia. It also assesses the factors that influence the intention to use e-consultations among the population. The use of e-consultations will ensure that healthcare services are accessible to all, reduce unnecessary hospital visits, and reduce long waiting times and crowding in hospitals. Exploring the factors influencing patients' use of e-consultations will help healthcare providers plan, improve, and sustain services to suit the users' needs (22). Additionally, the results of this study can be used as a baseline to monitor potential areas for improvement to increase the utilization of digital health.

## Methods

### Study Design

This study used a cross-sectional quantitative design. Patient perception and attitude toward utilizing online consultations was measured using a validated questionnaire informed by the technology acceptance model (TAM) (20, 23). The TAM has two dimensions, perceived ease of use and perceived usefulness, which influence the intention to use, and which might predict actual usage behavior (24). In this study, e-consultations were defined as the use of any remote medical consultation method (e.g., video, text, voice, or all). Participant consent was acquired, and the definitions of terms were described at the beginning of the questionnaire.

As the native language in Saudi Arabia is Arabic, the questionnaire was distributed in two languages (Arabic and English). Both content validity and face validity were assessed for both versions of the questionnaire. The content validity of the questionnaire was assessed using an expert panel review. Arabic-speaking academic experts from Imam Abdulrahman Bin Faisal University (IAU) and physicians from King Fahd Teaching Hospital (KFTH) were invited to assess the content validity of the questionnaire. A total of 12 experts participated online between February and March 2020 to review both the English and Arabic versions and verify their semantic equivalence.

To assess face validity, a purposive sampling technique was used to sample 25 members of the target population. Face validity was determined via one-on-one interviews. To indicate maximum variation, potential participants were selected from different age groups, levels of education, and genders. The internal consistency reliability of the scale was tested using Cronbach's alpha, and the scale was found to be reliable (overall Cronbach's α ¼ 0.868). The reliability of specific domains within the scale was tested, and for all the domains, the Cronbach's α coefficients ranged from 0.756 to 0.905.

### Recruitment Strategy

After the assessments of content and face validity were carried out, the online questionnaire was designed on Question Pro and distributed online via social media platforms, mainly WhatsApp and Twitter, using a convenience snowball sampling method. The inclusion criteria were being an adult aged 18 and above, living in Saudi Arabia, and speaking English or Arabic. The inclusion criteria were applied by adding questions related to age, language, and city of residency in Question Pro. The research team excluded respondents who did not meet the research criteria. Distributing the current study questionnaire via online channels was appropriate given both the aim of our research and the COVID-19 pandemic restrictions implemented during the data collection phase, which prohibited people from accessing hospitals, except in the case of emergencies. Recruitment was conducted from August to December 2020. Recall bias could have been introduced, as the data rely solely on the patient's recollection of their latest contact with an e-consultation service.

### Study Size

The sample size was estimated to be a minimum of 384 in accordance with the following formula:

$$\begin{array}{l} \text{N}=\frac{p\left(100-p\right)\;z^{2}}{E^{2}} \end{array}$$

where the ***p* =** population, which is estimated to be more than 1 million participants, ***e*** = margin of error is 0.5, and ***t*-value** is 1.96 (25).

### Study Questionnaire

**Figure 1:**
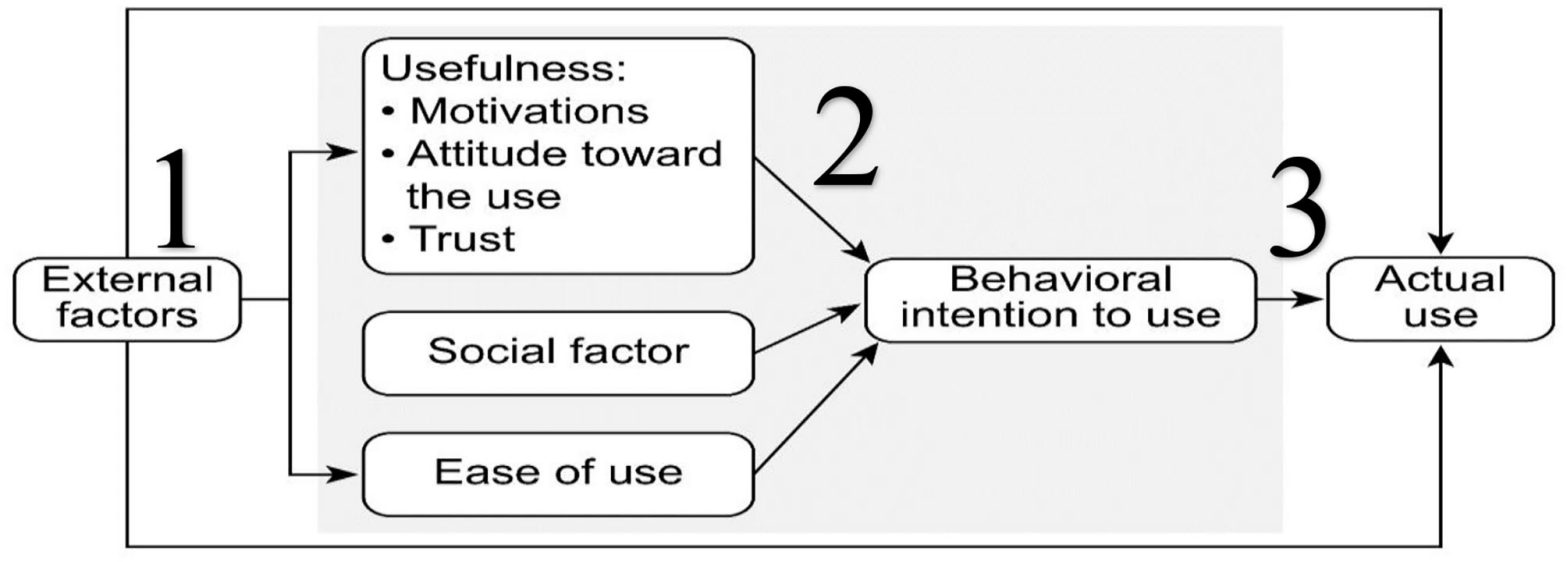
Model of theory.

Following the TAM model (Figure 1), the questionnaire included 25 items reported on a satisfaction scale (ranging from 1 = strongly disagree to 5 = strongly agree) (Appendix 1). External variables included age (as a continuous variable) and gender, area of residence, education level, occupation type, monthly family income, and the existence of a chronic disease (all as categorical variables).

### Statistical Analysis

The study analyses were performed in three stages. Stage 1: the external factors that influence the actual patient use of e-consultations were identified. These data were presented as descriptive and bivariate analyses. The external factors were patient social, economic, and clinical characteristics (categorical), and actual use of e-consultations was measured as “Yes” or “No.” Descriptive tests included the calculation of percentages, and the bivariate analysis and the Chi square test were used. Stage 2: the TAM factors (usefulness, social influence, ease of use) that influence the intention to use e-consultations were assessed as continuous outcomes. The relations were compared using the Pearson correlation test. Stage 3: the relation between intention to use (continuous variable) and actual use (categorical variable) was assessed. Before such an analysis was conducted, a normality assessment was performed using Shapiro–Wilk (Appendix 2) to explore the TAM factors (usefulness, attitude, social influence, trust, intention to use, and ease of use) and found to be not normally distributed. Therefore, non-parametric tests were used in the analyses. The significant results were reported at the 95% confidence interval, and all the analyses were performed using SPSS version 27.

## Results

The questionnaire was completed by 150 participants, with a response rate of 75%. Table 1 shows that the average age was 38 years old and 85% of the participants were females. The majority of the participants had a bachelor's degree (62%), worked in the government sector (39%), had a family monthly income that was more than SR20,000 (32%), and did not suffer from any chronic disease (82%). Most importantly, given the accelerated shift toward the use of e-consultations in the pandemic period, 45% reported actual use of e-consultations. There were no significant differences between the e-consultation users and non-users based on the study factors (age, gender, educational level, and family income) (Table 2).

**Table 1:** Study sample external factors.

**Characteristics**	***N* = 150**	**%**
**Age**	Mean = 38	SD = 14
**Gender**		
Male	22	15.0
Female	128	85.0
**Educational level**		
Diploma and below	24	16
Bachelor	93	62
Postgraduate	33	22
**Occupational level**		
Government	59	39.0
Semigovernment	8	5.0
Private sector	23	15.0
Not employed	60	40.0
**Monthly family income**		
Less than 5,000 SR	10	7.0
From 5,001 to 10,000 SR	34	23.0
From 10,001 to 15,000 SR	34	23.0
From 15,001 to 20,000 SR	21	14.0
More than 20,000 SR	51	34.0
**Do you suffer from any chronic diseases?**		
Yes	35	23.0
No	115	77.0
**Have you used e-consultations?**		
Yes	67	45.0
No	83	55.0

**Table 2:** The influence of external variables between actual e-consultation users and never-users.

**Characteristics**	**Have you used e-consultations?**						
	**Yes**		**No**		**Chi^**2**^**	**df**	***P*-value**
	***N* = 67**	**%**	***N* = 83**	**%**			
Age^*^	40 (14)		37 (14)		0.147	1	0.701
**Gender**							
Yes	9	13.4	13	15.7			
No	58	86.6	70	84.3			
**Educational level**							
Diploma and below	11	16.4	13	15.7	0.311	2	0.856
Bachelor	40	59.7	53	63.9			
Postgraduate	16	23.9	17	20.5			
**Occupational level**							
Government	33	49.3	26	31.3	5.477	3	0.140
Semigovernment	3	4.5	5	6.0			
Private sector	10	14.9	13	15.7			
Not employed	21	31.3	39	47.0			
**Monthly family income**							
Less than 5,000 SR	2	3.0	8	9.6	5.315	4	0.256
From 5,001 to 10,000 SR	19	28.4	15	18.1			
From 10,001 to 15,000 SR	15	22.4	19	22.9			
From 15,001 to 20,000 SR	11	16.4	10	12.0			
More than 20,000 SR	20	29.9	31	37.3			
**Do you suffer from any chronic diseases?**							
Yes	17	25.4	18	21.7	0.282	1	0.596
No	50	74.6	65	78.3			

* *Mean (SD)*.

Using the Pearson correlation test, we found that perceived usefulness factors, including motivation, trust, attitude, and social influence, were significantly associated with participants' intention to use e-consultations in the future (Table 3). In particular, participants with a higher level of perceived usefulness of e-consultations (Pearson 0.50, *p* < 0.001) and those with a higher trust score (Pearson 0.58, *p* < 0.001) reported a higher score for the intention to use e-consultations in the future. Ease of use had a minor non-significant relation with an intention to use e-consultations (Pearson −0.04, *p* 0.509).

**Table 3:** The relation between the TAM factors and the intention to use e-consultations among the total study population.

**TAM factors**	**Intention to use** ***N*** **= 150**	
	**Pearson**	***P*-value**
**Usefulness**	0.497	<0.001
Motivation	0.142	0.034
Trust	0.580	<0.001
Attitude	0.281	<0.001
**Social influence**	0.192	0.004
**Ease of use**	−0.044	0.509

As shown in Table 4, e-consultation actual users had higher scores for perceived usefulness (median = 50, IQR = 8) than actual non-users (median = 48, IQR = 7); *p* = 0.014. Similarly, e-consultation actual users reported a higher association between social influence and the ease of use that influenced such use (*P* < 0.01).

**Table 4:** TAM factors influencing the use of e-consultations among actual users and non-users.

**TAM factors**	**Use of e-consultations**				**Mann–Whitney *U*-test**	***P*-value**
	**Users (*****N*** **= 67)**		**Non-users (*****N*** **= 83)**			
	**Median (IQR)**	**Mean rank**	**Median (IQR)**	**Mean rank**		
**Perceived usefulness**	50 (8)	83.11	48 (7)	69.36	2467.0	0.014
Motivation	12 (4)	77.68	12 (2)	73.74	2634.5	0.135
Trust	20 (5)	83.26	20 (3)	69.23	2471.0	0.057
Attitude	17 (3)	80.12	16 (2)	71.77	2260.5	<0.001
**Social influence**	10 (4)	81.70	9 (4)	70.49	2270.5	0.012
**Ease of use**	11 (5)	80.18	7 (4)	71.72	1391.0	<0.001
**Intention to use**	12 (3)	96.24	12 (1)	58.76	2365.0	0.002

## Discussion

This article identifies the factors influencing the actual use of e-consultations during the pandemic in Saudi Arabia and assesses the TAM factors that influence the intention to use e-consultations among the population.

Saudi Arabia has allocated a very large budget to accelerate the implementation of electronic services in the healthcare sector. However, some authors have reported that the transition to electronic services in the Saudi health system was very slow (17, 26). However, since the COVID-19 pandemic, the Saudi health system has witnessed a significant boost in digitalizing health services (15). Thus, it was crucial to identify the factors that influence actual use and intention to use such services. This study provides baseline information regarding actual users of e-consultations in the country. According to the literature, an increase in e-service utilization should ensure better access to care for all and achieve greater financial wealth and investment in the Saudi health sector (17, 22).

Further findings in the current study revealed that participants' trust in and perceived usefulness of e-consultation was significantly associated with their intention to use e-consultation services in the future. Many studies have found that trust in e-consultations is a major driving force for patients' adoption of e-consultation services (27, 28). Trust is a complex issue that might hinder the adoption of e-consultation because there is a lack of face-to-face interaction to promote patients' belief in doctors' reliability and ability to provide professional services (28). Thus, to enhance patients' trust in e-consultations, which will ultimately improve their adoption of e-consultations, research studies recommend fostering the social ties between patients and physicians (i.e., interpersonal trust) and the technological capabilities of the system that provides e-consultation services (i.e., technological trust) (28). This can be applied by establishing a robust review and a reporting system to allow the patients to report their satisfaction with the services offered during their e-consultations and thus enhance potential patient acceptance and adoption of the service (28). Additionally, eliminating the privacy and performance risks of e-consultation platforms will play a key role in enhancing patients' trust in e-consultations (28). The sharing of personal data through online platforms is a concern of users. Therefore, to ensure trust, it is important to use highly secured platforms for e-consultations and to provide health providers with adequate training on how to keep patients' data highly confidential during and after the e-consultation (29). Additionally, the standardization of telemedicine/e-consultations merits attention during and after COVID-19 (30).

Some studies have been conducted in Saudi Arabia to assess the uses of e-consultations, and they agreed that there was variation in the uses of e-health services, such as e-consultations, by region (17, 31–33). They found that people from western provinces had the lowest rate of using the Seha application as a type of consultation tool (32). However, the availability of e-services was found to be relatively high in western provinces of Saudi Arabia compared with other provinces (17). However, the uses of these services are lower than those in other provinces, as some of the published literature has found (33). This could be explained by the low awareness of online health services by the community of the western region, as suggested by another study (33). Western provinces have more rural areas, and previous literature has shown that people in rural areas are less likely to use e-services and prefer face-to-face communication with physicians (31, 34). The area of residence is an important factor among the study target population, as found in the literature; however, due to the disproportionality of respondents and limited sample size by area of residence, the current study could not draw any conclusion due to the high risk of selection bias.

Age could be a key factor in determining the rate of using services or telemedicine, and younger age groups might be more familiar with technology than older people. The difference in the mean age of users and non-users in the current study was very close; they were both between 37 and 40; however, users were from the older group. This is consistent with a previous study that found that although younger people are more aware of services such as the Seha application, they are less likely to use it than older people. The study concluded that the younger group might have good health and were less likely to use services (31). Additionally, for a more detailed comparison among the older population, it was found that people aged 30–39 had the highest odds of using 937 (a phone consultation service) than people aged 60 and older, especially if they had children (34). However, people aged above 60 might not be familiar with these services or they might not know how to use them. The reason for the discrepancies in the use of e-consultations by age group is still not clear. Further studies with qualitative analysis need to be conducted to identify the reasons for not using e-consultations among populations aged above 60.

An interesting finding in our study was that social influence, which another study referred to as the chances that the use of e-consultations would affect the way others think about the user (35), had a significant association with actual e-consultation use. This contradicts an earlier study that revealed that social risk is only a minor component of a perceived risk that hinders the adoption of e-consultation (24). One explanation of this contradiction is the unique culture of Saudi Arabia, where people pay significant attention to society and how others think of them (36). Thus, it is especially important to raise awareness of e-consultation among the public in Saudi Arabia to help them understand its benefits and how it works. Some research studies have suggested that the most prominent reason for the lack of e-consultation use is that the public is not aware of the existence of such services (37, 38). With the improvement of awareness, the public will think it is a reasonable choice to use e-consultations and will not make a negative judgment about e-consultation use. In turn, these services will be used more effectively.

## Limitations

This is one of few studies to explore the use of e-services in the Saudi context. However, possible limitations include the study's reliance on participant reports of their e-consultation utilization. In future studies, it is recommended that the actual utilization of e-consultations be assessed using the hospital reporting system. In addition, the study used a convenient non-probability sampling technique. This technique was chosen due to the difficulty of visiting hospitals with the precautionary measures for COVID-19. Another limitation is the small sample size, which might not represent the Saudi population. The study aimed to target more than 384 participants. However, evidence from the literature stated that a sample size of more than 200 participants is considered good and could yield valid results (39). Finally, the majority of participants lived in the Eastern Province. This could hinder the generalizability of the study findings to all other regions in Saudi Arabia.

## Conclusion

This study is unique in its use of a theoretical-based model to identify factors related to e-consultation use in Saudi Arabia. The study found that approximately 40% of study participants used e-Consultations during the period of rapid transition during the COVID-19 pandemic. Participants' trust in and perceived usefulness of e-consultations were significantly associated with their intention to use e-consultation services. Furthermore, the availability of e-services and awareness of using services could be affected by where users live. Policymakers' attention to those factors could play a role in increasing public acceptance and the use of e-consultations to improve distance medical care.

## Data Availability Statement

The raw data supporting the conclusions of this article will be made available by the authors, without undue reservation.

## Ethics Statement

The studies involving human participants were reviewed and approved by Imam Abdulrhaman Bin Faisal University. The patients/participants provided their written informed consent to participate in this study.

## Author Contributions

ArA, BH, DA, and AfA contributed to the design and implementation of the research, the analysis of the results, and the writing of the manuscript. All authors contributed to the article and approved the submitted version.

## Conflict of Interest

The authors declare that the research was conducted in the absence of any commercial or financial relationships that could be construed as a potential conflict of interest.

## Publisher's Note

All claims expressed in this article are solely those of the authors and do not necessarily represent those of their affiliated organizations, or those of the publisher, the editors and the reviewers. Any product that may be evaluated in this article, or claim that may be made by its manufacturer, is not guaranteed or endorsed by the publisher.

## Abbreviations

TAM: Technology Acceptance Model
KFTH: King Fahd Teaching Hospital
IAU: Imam Abdulrahman Bin Faisal University.
