# Supplementary material for: Patient Acceptance and Intention to Use e-Consultations During the COVID-19 Pandemic in the Eastern Province of Saudi Arabia
Source: Front Public Health. 2022 Jun 30;10:896546. doi: 10.3389/fpubh.2022.896546 (PMC9280353; doi:10.3389/fpubh.2022.896546)
Supplement: Supplementary file 1 [file Table_1.docx]

**Appendices**

**Appendix 1** Study Questionnaire

| Dear Participant, We are a group of researchers at the College of public health at Imam Abdulrahman Bin Faisal University.  We are conducting this study to learn more about the patients’ perception of electronic consultation (e-consultation) during COVID19 in KSA from March to August 2020.  The information obtained in this survey will be used for research purposes only. It will be completely anonymous and confidential, and not shared with any other organization.  By submitting this form you are indicating that you have read the description of the study, are over the age of 18, and that you agree to the terms as described. The survey should take about 7 minutes to complete.   Thank you in advance for your time. | | |
| --- | --- | --- |
| **1.Demographic Data** | | |
| **1** | **Region** | 1.Makkah 2.Riyadh 3.Eastern Province 4.Aseer 5.Jazan 6.Almadinah Almonawarah 7.Qassim 8.Tabulk 9.Hail 10.Najran 11.Aljawf 12.Albaha 13.Northern Border |
| **2** | **Age** | Continues |
| **3** | **Gender** | 1.Male 2.Female |
| **4** | **Nationality** | 1.Saudi 2 Non-Saudi |
| **5** | **Education level** | 1.High School 2.Diploma 3.Bachelor 4.Post Graduate 5.Other, please specify |
| **6** | **Occupation** | 1.Governmental Sector 2.Semi-Government Sector 3.Private Sector 4.Self-employed 5.Not Employed |
| **7** | **Monthly family income** | 2.1.Less than 5,000 SR 3.From 5,001 to 10,000SR 4.From 10,001 to 15,000SR 5.From 15,001SR to 20,000SR 6.More than 20,000SR |
| **8** | **Do you suffer from any chronic diseases** | 1.Yes 2.No |
| **9** | **Have you used e-consultation** | 1.Yes 2.No |
| **2.Perceived Usefulness (Motivation, Trust, Attitude), Social influence, Intention to use and Ease of use questions** | | |
| Number | Questions/Item | |
| **1** | To what extent did the below factors enhance your utilization of e-consultation: | To be able to contact a physician about my health concerns at any time |
| **2** |  | To save on travelling time |
| **4** |  | To be able to ask questions that might arise after a visit to the physician |
| **5** |  | To seek second opinion |
| **6** |  | To ask how I can best cope with my health problem |
| **7** |  | To ask questions about medication use (for example side effects) |
| **8** |  | To help relieve stresses and worries about my symptoms |
| **9** |  | To reduce my uncertainty |
| **10** |  | To decide whether a visit to the physician is necessary |
| **11** |  | To improve my wellbeing (motivation) |
| **12** | To what extent do you agree with the following: | The treating physicians in the e-consultation services are honest |
| **13** |  | I believe that the health service provided by e-consultations platform is useful |
| **14** |  | Physicians on the e-consultations platform have medical qualifications. |
| **15** |  | The consultation or diagnosis provided by doctors on e-consultations platforms is reliable |
| **16** |  | In my opinion, physicians on the e-consultations platform are trustworthy. |
| **17** | To what extent do you agree with the following | Family and friends influence my decision of using the e-consultations platforms. |
| **18** |  | Social media influencers influence my decision of using the e-consultations platforms. |
| **19** |  | National policies and laws influence my decision of using the e-consultations platforms. |
| **20** | To what extent do you agree with the following: | I intend to use e-consultations platforms to consult health issues when needed in the future. |
| **21** |  | I plan to use e-consultations platforms to consult health issues when needed in the future. |
| **22** |  | I am welling to explore new applications/system for online consultations |
| **23** | In your opinion, what are the factors that would negatively affect using e-consultation? | The use of the Internet and e-mail is difficult |
| **24** |  | The application/software used for the online consultation is difficult to use |
| **25** |  | Not owning a smart phone or home internet network |
| **26** |  | Worried about smart phone and internet bills |

**Appendix 2:** Normality Assessment

| **Tests of Normality** | | | | | | | |
| --- | --- | --- | --- | --- | --- | --- | --- |
|  | **Have you used e-consultation?** | **Kolmogorov-Smirnov^a^** | | | **Shapiro-Wilk** | | |
|  |  | **Statistic** | **df** | **Sig.** | **Statistic** | **df** | **Sig.** |
| **Age** | **Yes** | 0.148 | 89 | 0.000 | 0.916 | 89 | 0.000 |
|  | **No** | 0.15 | 133 | 0.000 | 0.912 | 133 | 0.000 |
| **Gender** | **Yes** | 0.509 | 89 | 0.000 | 0.437 | 89 | 0.000 |
|  | **No** | 0.509 | 133 | 0.000 | 0.437 | 133 | 0.000 |
| **Area of residency** | **Yes** | 0.421 | 89 | 0.000 | 0.678 | 89 | 0.000 |
|  | **No** | 0.373 | 133 | 0.000 | 0.728 | 133 | 0.000 |
| **Education level** | **Yes** | 0.299 | 89 | 0.000 | 0.788 | 89 | 0.000 |
|  | **No** | 0.343 | 133 | 0.000 | 0.749 | 133 | 0.000 |
| **Occupation Type** | **Yes** | 0.296 | 89 | 0.000 | 0.739 | 89 | 0.000 |
|  | **No** | 0.285 | 133 | 0.000 | 0.743 | 133 | 0.000 |
| **Monthly family income** | **Yes** | 0.181 | 89 | 0.000 | 0.878 | 89 | 0.000 |
|  | **No** | 0.217 | 133 | 0.000 | 0.861 | 133 | 0.000 |
| **Social Influence** | **Yes** | 0.104 | 89 | 0.019 | 0.958 | 89 | 0.006 |
|  | **No** | 0.103 | 135 | 0.001 | 0.971 | 135 | 0.005 |
| **Motivation** | **Yes** | 0.166 | 89 | 0.000 | 0.866 | 89 | 0.000 |
|  | **No** | 0.161 | 135 | 0.000 | 0.952 | 135 | 0.000 |
| **Attitude** | **Yes** | 0.215 | 89 | 0.000 | 0.797 | 89 | 0.000 |
|  | **No** | 0.163 | 135 | 0.000 | 0.943 | 135 | 0.000 |
| **Trust** | **Yes** | 0.115 | 89 | 0.005 | 0.931 | 89 | 0.000 |
|  | **No** | 0.158 | 135 | 0.000 | 0.926 | 135 | 0.000 |
| **Intention** | **Yes** | 0.229 | 89 | 0.000 | 0.842 | 89 | 0.000 |
|  | **No** | 0.219 | 135 | 0.000 | 0.877 | 135 | 0.000 |
| **Ease of use** | **Yes** | 0.106 | 89 | 0.015 | 0.955 | 89 | 0.004 |
|  | **No** | 0.132 | 135 | 0.000 | 0.913 | 135 | 0.000 |
| **Usefulness** | **Yes** | 0.11 | 89 | 0.010 | 0.955 | 89 | 0.004 |
|  | **No** | 0.104 | 135 | 0.001 | 0.975 | 135 | 0.014 |
| **a. Lilliefors Significance Correction** | | | | | | | |
